# Biofortification of Sprouts and Microgreens with Trace Elements and Calcium

**DOI:** 10.3390/molecules31111860

**Published:** 2026-05-28

**Authors:** Magdalena Michalczyk

**Affiliations:** Faculty of Food Technology, University of Agriculture in Krakow, al. Mickiewicza 21, 31-120 Krakow, Poland; magdalena.michalczyk@urk.edu.pl

**Keywords:** mineral deficiency, microgreens, sprouts, plant enrichment

## Abstract

A large proportion of the world’s population struggles to meet the body’s requirements for certain minerals. It raises interest in methods of enhancing their levels in food raw materials. Sprouts and microgreens are highly promising raw materials for biofortification. Mineral accumulation in these raw materials may be influenced by genetic factors, as well as by concentration, form and method of mineral application, light conditions, and the plant growth period. In some cases, studies have reported several dozen-fold increases in the content of the applied mineral compared with control samples. However, in some experiments, selenium accumulation was so high that it may pose a risk to consumers. The topic of the human body’s ability to utilise the components supplied in this form has also not been sufficiently researched. There is a lack of publications on minerals such as chromium, magnesium, and copper. The research focuses mainly on biofortification with selenium, zinc, iron, calcium and iodine. Nevertheless, even for these minerals, the number of studies is still insufficient to develop precise biofortification protocols that take into account both the genetic characteristics of the plants and their growing conditions, so as to ensure an adequate supply of the missing minerals and consumer safety.

## 1. Introduction

Inadequate intake of essential nutrients may result in severe health complications. It is estimated that various forms of micronutrient deficiency may affect over 3 billion people [1]. Iron, zinc and calcium are among the minerals that are often consumed in deficient amounts [2,3,4]. Problems with insufficient supply of certain macro- and micronutrients may affect not only developing countries. A systematic review of 28 studies conducted among older adults in Western countries demonstrated a prevalence of zinc, selenium, iodine and copper deficiencies [5]. The issues indicated above have raised considerable interest in various methods of food fortification. This process can be carried out by increasing the accumulation of selected compounds in plants through soil or foliar fertilisation. Animal feed and food products themselves can also be enriched [6]. However, fortification of plants in fields may encounter certain difficulties. Haug et al. [7] point out that, for example, in the case of selenium added to fertilisers, only a small proportion is used by plants. Most of the unused selenium is lost, which is particularly worrying due to the limited global resources of selenium. When supplementing soil with iron, the problem is its binding to soil constituents and its low mobility, resulting in low efficiency [8,9]. The environmental impact of fertilisers and the difficulties associated with determining the optimal timing of fertilisation for effective enrichment of the edible parts of the plant are also important [10]. Therefore, it seems that sprouts and microgreens, which are characterised by a short growth period under fully controllable conditions, may be an attractive target for biofortification. Microgreens require a slightly longer growth period than sprouts. Unlike sprouts, they are cut above the growing medium and consumed without the roots. Microgreens are becoming increasingly popular and, like sprouts, can be grown not only industrially but also at home. Due to their sensory appeal and high content of nutrients and bioactive compounds, their potential application in astronaut nutrition is also being considered [11,12,13]. However, whether the content of a given component is higher or lower in unmodified microgreens than in mature vegetables depends, among other things, on the component and species being considered [14]. For example, Weber [15] found higher levels of P, Mg, S, Mn, Fe, Cu and Zn in lettuce and cabbage microgreens than in their mature counterparts purchased at the market. Khoja et al. [16] compared the mineral content of three types of mature vegetables purchased in a store and microgreens cultivated hydroponically. According to the results, mature fenugreek contained more Fe, Ca, Mg, Mn, and Mo and less Zn and Cu than microgreens, and mature rocket contained more Fe, Ca, Cu, Mg, and Mn and less Mo and Zn than microgreens. In contrast, mature broccoli contained similar Fe content and more Ca and Zn, and less Mg, Mn, and Mo than microgreens. However, studies conducted by these authors on iron uptake in Caco-2 cells revealed significantly higher values for microgreens than for mature fenugreek, whereas no differences were observed for rocket or broccoli. The authors highlight the need for human trials to assess iron absorption from microgreens, as well as the importance of factors such as endogenous iron absorption inhibitors, including phytic acid and polyphenols, and compounds that enhance iron bioavailability, such as ascorbic acid and fructose 1,6-biphosphate [16]. Similarly, in the case of trace elements other than iron, their bioavailability will be affected by the form in which they are found in the raw material and the presence of other compounds that may affect bioavailability, including fibre, phytates, polyphenols, possibly oxalates, and interactions between the minerals themselves related to, among other things, mutual competition [17]. It is also emphasised in the literature that microgreens require less space and water than mature vegetables [18]. However, the disadvantage of this production is the low weight of the product obtained from a single seed. Another disadvantage is the relatively short shelf life. The literature review presented below also indicates that, in some cases, biofortification of sprouts and microgreens can lead to very high concentrations of specific minerals. Biofortification exceeding nutritional requirements or insufficiently controlled may pose a risk to consumers.

## 2. Methods of Biofortification

Sprouts do not require substrates during cultivation, but the water used to soak the seeds or irrigate the sprouts can be additionally enriched. In the case of microgreens, in addition to the aforementioned treatments, various cultivation methods can be used: soil cultivation; soil substitutes such as peat, coconut, or jute fibres; hydroponic cultivation; and, rarely, aeroponics, where nutrient solution is sprayed onto the roots through an atomiser. In addition, attention is also drawn to the potential use of absorbing water or solution hydrogels, such as agarose hydrogels [19]. Significant differences were found between the mineral content of microgreens grown under different conditions. For example, lettuce and cabbage microgreens grown in vermicompost contained higher concentrations of most measured minerals than their hydroponically grown counterparts in most cases [15]. Using the same mineral concentrations in nutrient solutions under open-field and greenhouse conditions can lead to very large differences in the concentration of the biofortified component in plants [20]. When cultivating microgreens hydroponically, either water enriched with a selected mineral or a complete nutrient solution with an elevated concentration of selected minerals can be used. The effectiveness of biofortification is influenced by, among other things, the species and variety of the plant, the type and concentration of the mineral component, and the time of harvest. *Perilla frutescens* var. *crispa* f. viridis sprouts were found to have higher concentrations of minerals, except for potassium, than microgreens of this plant grown on a watered cotton bed [21]. Observations on the enrichment of two varieties of common wheat grown in hydroponic conditions at different concentrations of Fe, Zn, Mg, and Cr solutions (0–200 μg/g) in hydroponic media revealed the influence of both variety and harvest time (4 and 7 days) on the accumulation of minerals in sprouts, with higher concentrations of these components obtained in plants on the seventh day. In addition, a very strong correlation was observed between the mineral concentration in the media and microelement assimilation in sprouts. The presence of ions in the media had the greatest effect on zinc, whose content increased up to 29 times compared to the control, and the smallest effect on magnesium, whose content increased up to 1.4 times compared to the control. The authors obtained promising results in the case of chromium, which is rarely used in research. Its content increased from 0 μg/g dw in control samples to a range from 130 μg/g dw (50 μg/g medium, 4 days) to 538 μg/g dw (200 μg/g medium, 7 days) [22].

The mineral content of sprouts and microgreens can be influenced by manipulating the concentrations of available minerals and by altering light conditions. Among other things, a relationship has been observed between the wavelength of light used in the cultivation of basil, beet, and mustard microgreens and the accumulation of specific minerals in plants. Partial replacement of red LED light with green light (in a blue, red, and far red basal combination) caused an enhancement in mineral content that was dependent on the plant species. Distinct increases were observed, especially in beet microgreens, including zinc, manganese, iron, magnesium, calcium, and potassium. Basil was the least sensitive to the described modification of growing conditions, but even in its case, an increase in iron content was obtained [23]. Supplemental orange LED light used in addition to the basal combination of blue, red, and far red light resulted in an increase in Fe, Mg, and Ca content in kohlrabi, broccoli, and mizuna microgreens [24]. When using white LEDs with different photosynthetic photon flux densities, it was found that this parameter affects the mineral content of arugula microgreens, which achieve the highest iron content at the lowest light intensity [25]. As highlighted by Xu et al. [26], numerous publications emphasise the important role of photoperiod and light characteristics in the absorption and utilisation of essential elements by plants.

The above data indicate the highly complex nature of the process of shaping mineral content in sprouts and microgreens, and the high individualisation of the results, which depend on both genetic and environmental factors.

## 3. Biofortification of Sprouts and Microgreens with Minerals

### 3.1. Selenium

Selenium is not listed as indispensable for plants [27]. However, its effect on plant response to abiotic stress, as well as on plant growth and development, has been observed [28]. Puccinelli et al. [29] found an increase in the germination index in seeds of basil grown in a nutrient solution containing sodium selenate compared to seeds of basil grown without this component.

It is estimated that selenium deficiency affects approximately one billion people [30]. This element plays an important role in the body’s antioxidant protection and is involved in redox processes [31]. Cui et al. [31], based on an analysis of 20 cohort studies, observed that selenium biomarkers are inversely associated with mortality, including cancer and cardiovascular mortality. Selenium content in most areas ranges from 0.01 to 2 mg/kg, with selenium-deficient soil defined as having a content below 0.5 mg/kg. Plants growing on such soils contain too little selenium for the people who consume them. The opposite problem is excessive selenium content in some areas, which can reach as high as 13–49 mg/kg. [6]. Attempts to enrich sprouts and microgreens with selenium compounds have been the subject of numerous studies (Table 1). The possibility of biofortification of microgreens with selenium was evaluated both using a nutrient solution and in the form of a foliar spray. Tavan et al. [30] compared both methods for 14-day-old Toscano black kale microgreens. An increase in selenium content in the nutrient solution corresponded to higher levels in plants (10, 20, and 40 μM selenium in the solution corresponded to 163, 277 and 893 μg/g dw in the plant, respectively). However, an increase in selenium content using the foliar application method did not result in the same increase (21, 14 and 24 μg/g dw in the plant). Supplying selenium to plants in the form of a nutrient solution was therefore much more effective than in the form of foliar spraying. At the same time, the authors did not find a significant effect of selenium solutions on fresh microgreen yield in both cultivation methods compared to the corresponding control samples. Sodium selenate at various concentrations was also applied in the hydroponic cultivation of basil, cilantro, and scallion microgreens [32]. When 80% of the seeds had germinated, Na_2_SeO_4_ was added to the nutrient solution (Table 1). The selenium content in plants increased with increasing solution concentration. Scallion showed the highest accumulation of this mineral, followed by basil and, least of all, cilantro. At the same time, in the case of cilantro, no significant effect on plant yield was observed, while in the other cases, yield decreased with increasing doses of the additive. The differences were not always statistically significant. The effect on the content of other elements depended on both the plant species and the selenium concentration. Increases and decreases in their content were observed, as well as cases with no significant differences between individual product variants. For scallions, additional cultivation was carried out using a nutrient solution with a Se concentration of 10 mg/L. The resulting plants contained over 300 μg Se/g fw. This indicates that the doses of this element in microgreens cultivation must be carefully selected, as concentrations in plants may reach toxic levels. Islam et al. [33] used sodium selenite (Na_2_SeO_3_) solutions to biofortify white winter wheat microgreens grown for 10 days in the DFT hydroponic system. In addition to the effect of the solutions on selenium content, they also observed a decrease in microgreen yield, which was dependent on selenium concentration (r = −0.955). The authors attribute the decrease in yield to the possibility of competition between selenium and essential ion transporters in conditions of nutrient deficiency. The selenium content in microgreens increased with increasing selenium concentration in the nutrient solution. Increases in the contents of carotenoids and total chlorophylls were also observed in selenium biofortified samples. For concentrations of 0.25 and 0.5 mg/L, there was an increase in the content of vitamin C, phenols and flavonoids. Still, these changes were not consistently statistically significant. The reason given by the authors for the increase in phenolic compounds is abiotic stress caused by the concentrations of selenium solutions used. The observations from the above-mentioned study are not fully consistent with those reported by Viltres-Portales et al. [18]. In addition to wheat, the authors also cultivated kale and kohlrabi microgreens. A mixture of sodium selenite and sodium selenate was used because these two forms of selenium differ in their ease of transport from roots to shoots and their rate of metabolism into bioavailable organic forms in plants [18]. The authors did not observe any effect of the solutions on yields, which did not differ significantly from those of control samples. They explain this by plants’ ability to convert inorganic selenium compounds into dimethyl selenide and dimethyl diselenide, which are volatile and much less toxic. No effect of the added compounds on total chlorophyll content and total polyphenolic compounds content was observed, while it significantly increased the total carotenoid content in kohlrabi. The solution used increased the selenium content in plants to an extent dependent on their species. Brassica species are known for their ability to accumulate large amounts of both sulphur and selenium, the latter of which replaces sulphur in biochemical systems. Among the factors influencing selenium accumulation in plants, apart from genetic factors related to both accumulation capacity and resistance to high Se concentrations, the authors mention the form of selenium supplied, the duration of exposure to the administered compounds, and the nutrient content of the medium used [18]. The type of light used during cultivation may also be important for the content of individual minerals in microgreens [23]. A mixture of sodium selenite and sodium selenate was also used in the cultivation of green pea, red radish and alfalfa microgreens [34]. The plants did not receive any other minerals during hydroponic cultivation. As a result of selenium biofortification, the content of this element in plants has increased (Table 1). The effect on the other quality characteristics studied (chlorophylls, carotenoids, phenolics, sugars and mineral content) varied depending on the plant species and the characteristics studied. For example, total chlorophyll content increased in green peas and decreased in alfalfa under the influence of selenisation. In many cases, the content of minerals such as Mg, Ca, K, Fe, Zn, Mn, Cu, and Mo did not differ significantly between selenised and non-selenised microgreens of a given species. No significant effect of selenisation on dry biomass (g per cup) was found either.

Sodium selenate was supplied as a nutrient solution during the soilless cultivation of coriander, green basil, purple basil and tatsoi microgreens [35]. In the case of coriander and tatsoi, product yield increased with increasing selenium concentration, whereas in green and purple basil, no significant differences were observed. Interestingly, the addition of selenium to the nutrient solution reduced the nitrate content in microgreens. The content of other minerals was modified differently by the addition of selenium, depending on the plant species and the specific mineral. In the case of tatsoi, the content of P, K, Ca, Mg, Na, Fe, Zn and Mn decreased. In purple basil, the content of all minerals increased, except for Na. In green basil, Zn content decreased by more than 50%, whereas the contents of P, K, Ca, Mg, Na and Mn increased. In coriander, the content of all minerals tested increased, although at lower selenium concentrations, often insignificantly. The addition of selenium to crops caused an increase in the content of this element in plants in a dose-dependent manner. The highest selenium accumulation was found in green basil, followed by purple basil and tatsoi, with the lowest in coriander. The assessment of the effect of the enriching ingredient on the content of β-carotene and lutein does not provide a clear picture. Only in the case of tatsoi was an increase in these bioactive components observed; in the other cases, results varied and depended on both the plant species and the dose. Similarly, the effect of biofortification on polyphenol content was ambiguous [35]. An attempt was also made to enrich microgreens by pre-soaking mizuna, arugula, green basil, cress and radish seeds [36]. The yield of selenium-enriched microgreens was higher than that of controls, but this difference was not significant except for radish. Pre-soaking significantly increased the selenium content in plants (Table 1). However, selenisation also affected the content of other minerals in plants, with the effect varying significantly depending on the species. For example, selenisation caused an increase in Ca and Fe content in cress and a decrease in the content of these minerals in mizuna, arugula and radish compared to the controls. As a result of pre-soaking green basil seeds in a selenium-containing solution, the levels of all tested minerals increased in the microgreens obtained compared with the controls. The effect of selenium biofortification depends on the form of the element, but germination temperature may also be important. In the cultivation of lupin sprouts, higher selenium accumulation was found when selenate solution was used than when selenite solution was used. Furthermore, at certain concentrations (4 and 8 mg/L of selenate), the selenium content in plants was higher when germination was carried out at 20 °C than at 25 °C [37]. An interesting method of enriching basil microgreens with selenium was described by Puccinelli et al. [29]. In the first part of the experiment, basil was grown in separate hydroponic systems, with plants irrigated with a nutrient solution containing 0, 4 or 8 mg Se/L as sodium selenate. The seeds obtained in this way were used to grow microgreens containing 0, 117, and 203 μg Se/g dw, respectively.

The above examples, along with those in Table 1, indicate the possibility of a very significant increase in the selenium content of sprouts and microgreens. However, this element has a narrow therapeutic index and exhibits strong toxicity at doses above 700 μg/24 h [38]. Therefore, it seems necessary to establish precise protocols covering plant species and varieties, as well as the specific growing conditions required to obtain selenium-enriched raw material that does not pose a health risk even when consumed regularly in high doses. These species, which accumulate very high concentrations of selenium, could potentially serve as raw material for the production of dietary supplements. The form in which selenium, biofortified in various protocols, is found in plants and its availability for use by the human body requires separate assessment. Other ingredients may also have an influence, for example, according to Frączek and Pasternak [38], the presence of vitamins A, D, and E has a beneficial effect on selenium absorption.

**Table 1:** Results of selenium biofortification of sprouts and microgreens.

Raw Material	Treatment	Results	Reference
Radish sprouts	Sprouts were cultivated in sodium selenite (Na_2_SeO_3_) solution (0, 1, 2, 5, 10 mg/L)	1, 2 ↑ growth status; 5, 10 ↓ growth status; similar concentration of Se in stem and leaf, higher in root; at 5 and 10 mg/L, Se content in stems was 19.5 and 51.6 times higher than that of the control group	[39]
14-day Toscano black kale microgreens	Two Se (as Na_2_SeO_4_) application methods were assessed: supplementation into the nutrient solution or as a foliar spray at concentrations: 0, 10, 20 and 40 μM Se	Microgreens accumulated up to 893.3 (nutrient solution) and 24 (foliar treatment) μg Se/kg dry matter compared to control (1.8 and 0.6 μg Se/kg dry matter); yield was unaffected.	[30]
Wheat microgreen extract	DFT hydroponic system with concentrations of Se (0, 0.125, 0.25, 0.50, and 1.00 mg/L from sodium selenite	↑ selenium content with increasing Se concentration in cultivation	[33]
Kale, kohlrabi, wheat microgreens	0 or 20 μmol/L of a mixture of Na_2_SeO_3_ and Na_2_SeO_4_ (1/1, *v*/*v*) in tap water solution applied twice a day	133 μg Se/g dw in kale, 127 μg Se/g dw in kohlrabi, 28 μg Se/g dw in wheat; in controls less than 1–2 μg Se/g dw, no effect on biomass	[18]
Mizuna, arugula, green basil, cress, radish microgreens	Soaking seeds (30 min) in 2 mg Se/L solution (as sodium selenate)	Se content from 0.07 to 0.26 µg/g dw (control) and from 71.9 to 132.5 µg/g dw (fortificated plants)	[36]
Coriander, green basil, purple basil, tatsoi microgreens	0, 8, or 16 µM of sodium selenate in the nutrient solution	Se content (0, 8, 16 µM): coriander 0.05, 8.6, 26.2; green basil 1.1, 67.4, 150; purple basil 3, 50.1, 109.3; tatsoi 0.04, 21.2, 61.3 µg/g dw	[35]
Amaranth microgreens	Seeds were primed in 25 or 100 ppm selenium nanoparticles (SeNPs) solutions (60 min); 100 ppm SeNPs solution in soil application; 25 ppm for foliar spraying	↑ fresh and dry weight and selenium content 0.6 (control), 4.5 (seed priming 100 ppm), 8.2 (seed treatment and soil application), 10.7 mg/g (seed treatment, soil and foliar application)	[40]
25-day basil, cilantro, scallions microgreens	Na_2_SeO_4_ in nutrient solution (0, 2.5, 5 mg/L of Se and 10 mg/L of Se for scallions)	↑ Se content up to 2481.4 μg/g dw of scallions (10 mg/L)	[32]
Broccoli sprouts	100 µmol/L selenite Na_2_SeO_3_ or selenate Na_2_SeO_4_ in spraying water	the total Se contents in the selenite- and selenate-treated sprouts were 75 µg/g and 85 µg/g dw, respectively. The controls did not contain Se.	[41]
Broccoli, cauliflower, green cabbage, Brussels sprouts, Chinese cabbage, kale sprouts	0 or 50 µM Na_2_SeO_4_ during growth	Se was not detectable in control sprouts. In treated sprouts Se content was most often about 160 µg/g dw, but in Brussels sprouts, it was less than about 70 µg/g dw	[42]
4-day chickpea sprouts	Soaking 100 g seeds (6 h) in Na_2_SeO_3_ solutions (85 mL water containg 0, 1 or 2 mg Na_2_SeO_3_)	Se content: 0.06, 3.54, 6.93 µg/g dw of sprouts) for 0,1 and 2 mg Na_2_SeO_3_	[43]
*Amaranthus cruentus*, *A. caudatus*, *A. paniculatus*, *A. tricolor* sprouts	Seeds soaking in 0, 10, 15, 30 mg Se/L as sodium selenite solutions (3 h)	Controls: 0.3 to 1.4 mg Se/kg dw; 15 mg /L—content in sprouts: from 45 to 82 mg/kg dw; 30 mg/L causes a reduction in plant development	[44]
Red radish, green pea, alfalfa microgreens	Sodium selenite and sodium selenate (1:1) at a total concentration of 20 μM for watering solution in a hydroponic vertical farming system	Se content in treated plants was 43−70 mg Se/kg dw; in controls less than 1 mg Se/kg dw; ↑ chlorophylls and carotenoids in green pea, ↓ chlorophyll levels in alfalfa, and ↔ in red radish	[34]
Lupin sprouts	Na_2_SeO_3_ and Na_2_SeO_4_ (2, 4, 6, and 8 mg/L) during germination	Se content 0.14 (μg/g dw) for control, up to 4.9 (μg/g dw) for Na_2_SeO_3_ solution, up to 13.5 (μg/g dw) for Na_2_SeO_4_ solution	[37]
Broccoli microgreens	Se (100 μmol/L Na_2_SeO_3_) in spraying solution, UVA (40 μmol/m^2^/s) and Se	↑ organic Se content; Se UVA inhibited microgreens growth and ↑ some phytochemical contents	[45]
10-day rice sprouts	(15, 45, 135, and 405 mg Se/L) of sodium selenite and sodium selenate	↑ organic and inorganic Se content and phenolic acids content	[46]

Note: ↑ increase in content; ↓ decrease in content; ↔ no effect or result not significantly different from control. Abbreviations: Deep Flow Technique (DFT); dry weight (dw); selenium nanoparticles (SeNPs).

### 3.2. Iodine

Iodine is essential for the production of thyroid hormones. It is estimated that approximately two billion people are affected by iodine deficiency. Despite efforts such as salt iodisation programmes, the difficulty of fully meeting iodine requirements through diet alone suggests that research on iodine food supplementation remains relevant [47,48]. The problems associated with supplementing iodine deficiencies by adding potassium iodide to table salt are, on one hand, iodine volatilisation during salt storage and, on the other hand, recommendations to limit its consumption [48]. One possible approach to increasing iodine intake in the diet is enriching microgreens with iodine. Such attempts using tatsoi, coriander, green basil, and purple basil seeds were conducted by Ciriello et al. [48]. They found the highest content of this component, 15 μg/g fw, in tatsoi irrigated with a nutrient solution containing 8 μM iodine as potassium iodide. In all tested species, compared with the control samples, the content of this component increased, with a greater increase after application of 8 μM iodine than after 4 μM. In another study, Wang et al. [49] drew attention to the effect of *Bacillus velezensis* (rhizobacteria) on iodine absorption in relation to microgreens of pepper (*Capsicum annatum* L. Tianjiao). The authors used two concentrations of KI solution for seed soaking: 0.01 and 1 mmol/L, with or without a mixture of *B. velezensis*. Interestingly, the highest iodine content in mg/kg d.w. of microgreens was obtained for the combination of 0.01 mmol/L KI solution and the *B. velezensis* mixture. Lower KI concentrations resulted in greater fresh biomass and greater colonisation by *B. velenzesis*, which, according to the authors, accelerated 0.01 mM iodine absorption. However, in all cases, the increase in iodine content was less than approximately three times that of the control. According to some works, trace amounts of iodine seemed to be essential for plant growth, affecting, among other things, photosynthetic efficiency and biomass accumulation, but excessive concentrations are toxic to plants [50,51]. It has been shown that iodine stress disrupted pathways associated with photosynthesis, cuticle biosynthesis and antioxidant defence in pepper leaves grown under soil conditions [51]. Similarly, Gorše et al. [52] found a lower germination rate in sprouts obtained after soaking Tartary buckwheat seeds in a potassium iodate solution (1500 mg I/L, 8 h) compared to the control. However, as Kiferle et al. [50] point out, the role of iodine in plant physiology is poorly understood, and to date, it is not mentioned in the list of plant micronutrients. Nevertheless, quite numerous studies have been conducted on the possibility of enriching vegetables in order to increase the amount of this element in the human diet, including kale, basil, spinach, carrots, Swiss chard and sea beet baby leaves [53,54,55,56,57]. In addition to increased iodine content in plants, the observations also included species-dependent changes in the content of other minerals [58], promoting early flowering [50], and positive or negative effects on biomass [59]. However, relatively few attempts have been made to enrich sprouts and microgreens with iodine (Table 2). The results are encouraging, although some studies did not find very high iodine accumulation. The highest results reported in Table 2 were obtained for legume sprouts. However, the highest result for white clover sprouts, whose seeds were soaked in water and irrigated with a potassium iodide solution, showed reduced biomass. On the other hand, red clover seeds soaked in iodine solution and watered with water or iodine solution yielded significantly higher biomass than in the control [60].

The insufficient number of studies on the possibility of enriching sprouts and microgreens with iodine does not allow for an assessment of which method, seed soaking or plant watering, is more effective. The upper limits of iodine solution concentrations well tolerated by plants, as well as the maximum concentrations of this element that they can accumulate, are also unknown. It also seems important to clarify the role that selected strains of microorganisms can play in iodine biofortification of sprouts and microgreens.

### 3.3. Zinc

Chasapis et al. [63] report that nearly 17% of people worldwide are affected by Zn deficiency. This trace element plays structural, catalytic, and regulatory roles, is a cofactor for over 300 enzymes, is involved in DNA synthesis and replication, and its deficiency can result in many different health problems, including immune system disorders. At the same time, the human body cannot store zinc, and it must be supplied daily [63]. In plants, zinc is necessary for proper growth and development and contributes to defence against pathogens, auxin production, photosynthesis, and other functions [64,65]. Excess zinc is also harmful to plants, causing, among other things, chlorosis and yield loss [66]. According to Sadeghzadeh, a study of 190 field trials in 15 countries worldwide (Sillanpää, 1990) found Zn deficiency in 49% of the trials [67]. Since zinc deficiencies can reduce the yield and quality of crops, ZnSO_4_, ZnO, ZnCO_3_, ZnCl_2_, and Zn(NO_3_)_2_ are used in soils poor in this element. However, the use of zinc as a fertiliser for field crops encounters various difficulties, both economic and related to, for example, the distribution of the added element in the soil. Factors such as soil pH and liming are also important. An increase in pH reduces the availability of zinc, while a decrease in pH increases it. Soil temperature, soil moisture, organic matter content, and other cations are also important. The process of Zn^2+^ uptake by plant roots occurs through various mechanisms: mass flow, root interception mechanisms, and diffusion [67]. At the same time, excess zinc, e.g., associated with pollution, can be toxic to plants. High concentrations of zinc can reduce plant germination, depending on the species and concentration assessed, and modify root system development by increasing branching and reducing primary root growth [68]. The zinc concentrations reported in the literature as required for the proper growth of most plants range from 15 to 200 mg Zn/kg dry weight [68,69]. At least 28 plant species have been described that can hyperaccumulate zinc at very high substrate concentrations, which is one of the possible strategies plants use to cope with zinc excess. In such cases, the zinc content in shoots can reach over 1% of dry weight [68]. Nevertheless, Di Gioia et al. [70] found only 0.21 (Garnet giant mustard) to 0.75 (Black oil sunflower) mg Zn/100 g fw in 17 microgreen species grown without adding microelements, with 15 not exceeding 0.4 mg/100 g fw. Xiao et al. [71] report similar contents for 30 commercially grown microgreens belonging to the *Brassicaceae* family: from 0.22 to 0.51 mg/100 g fw. Attempts to enrich sprouts and microgreens with zinc or other nutrients by increasing zinc concentrations during cultivation have been the subject of several studies (Table 3). Biofortification in sprouts and microgreens can be achieved through nutrient solutions, foliar treatment, and seed soaking in enriched solutions. Soaking seeds is a common practice before germination. However, it is associated with the possibility of mineral loss. It would appear that there are too few publications devoted to detailed analyses of the effects of this process on the macro- and micronutrient content of sprouts and microgreens. There are few studies available that assess the effect of soaking seeds of different plant species on the mineral content of microgreens or sprouts, although the phenomenon of loss of various substances during seed imbibition is well known [72]. The effect of the soaking process itself (distilled water, 20 h) and germination (72 h) on the zinc and iron content in three white sorghum varieties was assessed by Afify et al. [73]. After soaking, they observed iron losses of 28–40% and zinc losses of 14–27%. The authors explain the lower losses in the case of zinc by the fact that zinc and iron are bound to different molecules in different parts of the seeds. Germination caused iron losses of approximately 39% and zinc losses of 22 to 31%. At the same time, soaking the seeds reduced the phytate content by 24–32% and their germination by 25–35%. The final result of soaking and sprouting was an increase in the phytate-to-iron ratio and a decrease in the phytate-to-zinc ratio. However, measured under in vitro digestion conditions, the bioavailability of iron and zinc increased in both cases after soaking (15–21% and 9–11%) and after germination (17–21 and 12–18%). Zou et al. [74] also examined the effect of soybean soaking on mineral content. The authors point to the losses of Fe, Mn, Cu, Ca and Mg that occurred after soaking soybeans in water. In the case of calcium, these losses averaged 97%, whereas in magnesium, they were only about 4%. The use of zinc solutions for soaking, depending on their concentration, reduced the losses of copper, manganese and iron in the soaked seeds. Nevertheless, after seed soaking, the authors found an average of 41% manganese loss (for solutions) and 57% manganese loss for water, 24% (solutions) and 47% (water) for copper, and 9% (solutions) and 17% (water) for iron. The distribution of biofortified zinc in individual parts of the obtained sprouts varied. Separation of ZnSO_4_-treated soybean sprouts into edible (cotyledon and hypocotyl) and inedible (radicle) portions revealed that, compared to the control in the ZnSO_4_-treated samples (Table 3), the zinc content in the edible portion increased 2.2–11 times, and in the inedible portion 20–82 times [74]. A 12 h seed soaking in solutions of various zinc compounds (Table 3) was also used for sunflower and pea microgreens [75]. Higher zinc accumulation was found in sunflower microgreens than in pea ones. At the same time, the latter plant was sensitive to a decrease in Fe, Mn and Cu content under priming. The highest Zn accumulation efficiency was observed after the application of zinc sulphate and ZnO at 200 ppm, whereas Zn-EDTA proved ineffective (Table 3). The use of ZnSO_4_ solutions in both pea and sunflower microgreens resulted in a lower phytic acid/Zn molar ratio, which, according to the authors, suggests the higher bioaccessibility of the biofortified Zn. In the case of sunflower, a similar but weaker effect was also found for ZnO and Zn-EDTA. Çolak Esetlili et al. [76] evaluated the effect of different zinc doses in the cultivation of three microgreen species (Table 3) on their mineral content. The zinc content in all plant species increased with the increase in the content of this component in the nutrient solution, but the greatest change compared to the control was caused by a zinc concentration in the nutrient solution of 5 mg/kg. The zinc content in the broccoli, cauliflower and cabbage microgreens obtained was 242, 138 and 225 mg/kg, respectively. The maximum concentrations in cultivation are given in Table 3. The effect of the element in question on the concentrations of other minerals assessed depended on the species and concentration. For example, in broccoli and cauliflower, as the zinc concentration increased, the copper concentration decreased, but no such relationship was observed in cabbage. The highest iron concentration was obtained in cauliflower grown at 20 mg Zn/kg, and the lowest in broccoli grown at the same concentration. The authors estimate that the other analysed components (P, K, Ca, Mg, Fe, Cu and Mn) were at optimal levels (generally similar to or even higher than the control) for crops grown at 5 mg Zn/kg.

Another option for biofortification with zinc and iron is the use of combined bio- and nanofertilisers [77]. The nanofertilisers used by the authors were ZnO (77 nm) and γ-Fe_2_O_3_ (68 nm), which were functionalised with a *Pseudomonas* preparation. The functionalised nanoparticles were prepared by reacting zinc or iron nanoparticle dispersions with a bacterial suspension for 8h at 30 °C and 150 rpm. The authors observed both an increase in fresh weight under the influence of the applied agents and an enrichment of plants with iron and zinc. However, other experiments conducted by the authors, concerning the effect of the nanoparticles themselves, indicate the significant role of bacteria in this increase in content. Without their participation, Zn and Fe content were higher than in the controls but lower than with microorganism participation.

In addition to differences in the type of zinc compounds and their concentrations, the studies use different time regimes for introducing minerals during cultivation. For example, Kathi et al. [78] irrigated arugula microgreens starting on the fifth day after setup to seedlings with emerging cotyledons, using various solutions: deionised water, ascorbic acid solutions and ascorbic acid solutions with solutions of different salts, including ZnCO_3_. A very high increase in zinc content was found in samples irrigated with its solution compared to the control, 66.7 and 0.9 mg/100 g d.w., respectively. Di Gioia et al. [79] assessed the possibility of biofortification with iron and zinc in arugula, red cabbage and red mustard microgreens. The aforementioned micronutrients were introduced after complete germination, on the third day after sowing. The addition of zinc increased the levels of these components in the microgreens, but differences in concentrations of 0, 5 and 10 mg/L were not always statistically significant.

The examples cited, and data from Table 3, indicate difficulties in drawing consistent conclusions regarding the optimal conditions for zinc biofortification. This is due to the still insufficient number of experiments, which do not allow for a systematic review of the impact of a given factor, e.g., the type and timing of biofortification in the plant cultivation cycle, the type and concentration of compounds, or other cultivation factors such as light, temperature, availability and mutual proportions of other minerals.

**Table 3:** Results of zinc biofortification of sprouts and microgreens.

Raw Material	Treatment	Result	Reference
Arugula microgreens	DI, DI + AA (0.25%) + pH adjuster (KOH or NaOH or Ca(OH)_2_ or ZnCO_3_, 10 days of treatment, 14–15 days after sowing	0.9 mg/100 g d.w. (DI) 66.7 mg/100 g d.w (ZnCO_3_)	[78]
11-day arugula, red cabbage, red mustard microgreens	zinc sulfate heptahydrate (0, 5, 10, 20 mg Zn/L) in nutrient solution	↑ of Zn from 75% to 281% for 5 and 10 mg/L, no effect on yield	[79]
Broccoli, pak choi, kohlrabi and kale microgreens	ZnSO_4_ × 7H_2_O at 0 (control), 5, 10, and 20 mg/L in hydroponic cultivation	5 and 10 mg/L ↑ Zn in all microgreens, ranging from 1.4- to 1.8-fold compared to the controls, 20 mg/L ↑ 2- to 2.9-fold compared to controls	[80]
Pea and sunflower microgreens harvested 9 and 10 (pea) days after sowing	seed nutri-priming, with ZnSO_4_H_2_O, Zn-EDTA (14% chelated Zn), and Zn oxide nanoparticles (concentrations: 0, 25, 50, 100, and 200 ppm)	200 ppm ZnSO_4_ ↑ Zn in peas (126.1%) and in sunflower (229.8%); ↓ Fe, Mn, Cu in pea microgreens	[75]
Cauliflower, broccoli, cabbage microgreens harvested 20th day after germination	ZnSO_4_ (0, 5, 10, 20 mg/kg) in the nutrient solution (NS)	Zn content from 0 to 20 mg/kg in NS: broccoli 121–266, cauliflower 65–310, cabbage 58–267 mg/kg; no statistically significant effect on fresh weight, 10 and 20mg/kg ↓ K	[76]
Three cultivars of brown rice germinated for 24 h	Seeds soaked in 0, 25, 50, 100, 150, 200, 250 mg ZnSO_4_/L for 10 h, germinated for 24 h	↑ Zn content (different in different cultivars) with an increase in Zn concentration in soaking solution; 200 and 250 mg ↓ % germination and GABA content;	[81]
Pea sprouts	Seeds soaked in 0, 10, 20, 30, 40, 50, 60 mg ZnSO_4_/L for 24 h. or sprayed with the same dose every day.	↑ Zn content, Zn content for 60 mg/L was 169 mg/kg (soaking), 211 mg/kg (spraying)—357-fold and 448-fold to control; ↑fresh weight, ↑ chlorophylls content; (according to authors, optimal dose 40–50 mg/L)	[82]
Soybean sprouts	Seeds soaked for 8 h in ZnSO_4_ solutions (0, 10, 20, 30, 40, 50, 60, 80, 100 μg Zn/mL), 5 days germination under periodic spraying with ZnSO_4_ solutions	↑ Zn content in cotyledon and hypocotyl from 32 (control) to 102 (10 μg/mL) and 395 mg/kg dw (100 μg/mL); ↑ Zn content in radical from 0.07 (control) to 1.44 (10 μg/mL) and 5.8 g/kg dw (100 μg/mL).	[74]
12-day broccoli microgreens	Uncapped ZnO (77 nm) NPs, bacteria-capped ZnO NPs, uncapped ZnO NPs with biofertiliser and others applied on days 1, 6 and 9 of the experiment	The highest ↑ Zn content for uncapped ZnO NPs and biofertiliser; ↑ of Zn content for bacteria functionalised ZnO NPs, uncapped ZnO NPs, and uncapped γ-Fe_2_O_3_ NPs with biofertiliser	[77]

Note: ↑, increase in content; ↓, decrease in content; Abbreviations: deionised water (DI); ascorbic acid (AA); γ-aminobutyric acid (GABA); nanoparticles (NPs); dry weight (dw); nutrient solution (NS).

### 3.4. Iron

Iron is another trace element whose deficiency is quite common. This condition results in anaemia, impaired immune response, and delayed development. Groups particularly vulnerable to iron deficiency include women, especially pregnant women, and children under five [83]. Khoja et al. [16] report that iron deficiency affects about a third of the world’s population. In plants, iron plays a role in photosynthesis, is a component of many enzymes, participates in CO_2_ fixation, phytohormone synthesis, nitrogen metabolism, and its deficiency causes iron chlorosis and stunted root growth [84,85]. Excess iron can cause bronzing, blackening of the roots, discolouration of leaves and stems, wilting of shoots and growth retardation. As in the case of zinc, neutral and alkaline soils are not conducive to its availability to plants [85]. The iron content in seventeen microgreen species reported by Di Gioia et al. [70] ranged from 0.25 to 0.47 mg/100 g fw. For 30 microgreens of *Brassicaceae*, the range was 0.47 to 0.84 mg/100 g fw [71]. In contrast, the cultivation of chicory, Swiss chard, and black cabbage microgreens yielded 1.0 and 2.1 mg Fe/100 g fw. After germination, the plants were irrigated with half-strength Hoagland nutrient solution containing, among other nutrients, iron [62]. Biofortification of microgreens and sprouts with iron has been the subject of several studies (Table 4). In addition to the increase in iron content in plants depending on the concentration of this element in the nutrient solution, Di Gioia et al. [79] found that higher iron concentrations (40 mg/L) reduced fresh yield, seemed to be phytotoxic, and increased the accumulation of other minerals in arugula, red cabbage, and red mustard microgreens. In control samples, the authors observed 0.49–0.77 mg of iron/100 g fw, whereas in samples grown with 10 mg of iron/L in the nutrient solution, they observed 0.83–1.74 mg of iron/100 g fw. Frąszczak and Kleiber [86], biofortifying microgreens of purple kohlrabi, radish, pea and spinach using a nutrient solution, reported that both 10 and 20 mg/L of iron caused a significant increase in the content of this element in microgreens. Similarly, the possibility of increasing the iron content in sprouts/microgreens is also indicated by the work of Guardiola-Márquez et al. [77], Dębski et al. [87], Utthanontri et al. [88], Zielińska-Dawidziak and Singer [89], among others. However, the possible biofortification of sprouts and microgreens with iron requires not only consideration of the influence of genetic factors on the possibility of accumulating this element, but also a comparison of the possibility of supplying this component in mature vegetables and their young counterparts, as well as the bioavailability of iron in various plant raw materials. For example, Li et al. [90], after administering 100 mL per day of rice milk (control), sprouted soybean milk and soybean milk to anaemic adolescent girls for 6 months, observed a decrease in the rate of anaemia and an increase in haemoglobin and plasma ferritin concentration in the case of soy products, with a significant increase in sprouted soybean milk. However, according to the authors, the differences in the results obtained for both soy products were small.

Biofortification can aim to reduce the content of iron absorption inhibitors in plants, increase the iron content, and increase the content of compounds that favour iron absorption. Degradation of phytate occurs, among other things, during germination [91]. A reduction in the content of phytic acid, which acts as a chelator of metal ions and reduces their bioavailability, can also be achieved through genetic modification of plants. Methods used in cereals include the application of the CRISPR/Cas9 genome editing tool, as well as mutations induced by ethylmethanesulfonate (EMS) or gamma irradiation [92]. However, phytic acid acts not only as a metal cation reserve in seeds, but also in the stomatal closure mechanism in leaves and in resistance against pathogens, so reducing its content may have an adverse effect on plants [91]. In addition, this substance may also have a beneficial effect on the proper functioning of the human body. It has been suggested that it may have anti-inflammatory and anticancer properties [93]. Pongrac et al. [94] also noted that at comparable Fe concentrations and proportions of F^2+^, the lower phytate content in tartary buckwheat sprouts than in grains did not correlate with greater bioaccessibility of Fe to human Caco-2 cells.

Biofortification through increased iron content can be achieved by inducing overexpression of the iron storage protein ferritin in transgenic crops. However, such changes in plants growing in soils contaminated with heavy metals may affect the cadmium accumulation, for example [91]. Zielińska-Dawidziak and Singer [89] achieved ferritin overexpression in soybean sprouts using FeSO_4_ solutions. Ferritin iron content in soybean sprouts in their experiment increased from 1.8 mg/100 g d.m. (distilled water) to 123 mg/100 g d.m. (5 mM FeSO_4_) and 175 mg/100 g d.m. (25 mM FeSO_4_). In the case of Fe^2+^ content, the corresponding values were 4.3, 5.4, and 62.9 (mg/100 g d.m) (Table 4). The authors conclude that the environmental stress imposed on the crop by FeSO_4_ solutions was mitigated by the synthesis of ferritin, which protects cells against toxic levels of iron. Research indicates that phytoferritin can be considered an alternative source of iron in the diet to animal products [95]. According to Zielińska-Dawidziak [96], the main direction of biofortification of plants with this element is the enrichment of legumes and cereals, with legumes being richer in this element. Despite the observed increase in iron content in biofortified sprouts and microgreens (Table 4), based on the cited publications, it is difficult to determine whether iron-enriched sprouts and microgreens can better balance a diet deficient in this nutrient than mature plants. The bioavailability of iron from these products remains insufficiently researched. Another insufficiently explained issue is the impact of iron supplementation on the content of other minerals.

### 3.5. Calcium

Calcium is a component of bones and teeth and is involved in muscle contraction and nerve signalling. In some parts of the world, calcium deficiency is reported, whereas in others, excessive intake is also observed [97]. According to Thor [98], the calcium content in plant shoots ranges from 0.1 to over 5% of dry weight. It plays both a structural role and acts as an intracellular second messenger in many processes. The literature reports calcium contents in microgreens ranging from 29 to 477 mg/100 g fw, depending on the variety and cultivation method [70,71,99,100,101]. Calcium can be used both as an element enriching the nutritional value of sprouts and microgreens and as a compound extending their short shelf life [102,103]. Kou et al. [102] sprayed broccoli microgreens with CaCl_2_ solutions and found that the use of 10 mM not only resulted in an almost threefold increase in calcium content compared to the control, but also improved overall quality and reduced off-odour during 21 days of storage at 5 °C. The treatment also affected the activity of certain enzymes (Table 5) and the expression of senescence-associated genes. Abe and Oshita [103] point out that the calcium-enriched broccoli microgreens they used contained it as a Ca(II) hydrated complex, whereas in other plant materials, such as spinach, this mineral may occur as insoluble Ca oxalate, which hinders its absorption by the human body. Calcium can also affect the accumulation of other substances in plants treated with it (Table 5). For example, an increase in the content of total phenolics, phenolic acids and isoflavonoids in soybean sprouts [104], an increase in the content of resveratrol in peanut sprouts [105] and an increase in total phenolic content in common vetch sprouts treated with CaCl_2_ solution [106] were observed. In a study comparing the quality of aboveground sections of Chinese kale sprouts grown under different lighting conditions, with or without CaCl_2_ treatment, total phenolics increased with calcium treatment in white and red light but decreased in blue light. Total glucosinolates decreased under white light, while changes under other lighting conditions were insignificant. At the same time, 10 mM calcium chloride (CaCl_2_) solution reduced the levels of chlorophylls, carotenoids, flavonoids and soluble proteins in Chinese kale sprouts grown under white, red or blue light [107]. The example cited above illustrates the complexity of the interactions between factors determining plant growth conditions. According to research by An et al. [106], the form of calcium is also important for its accumulation in sprouts. The authors indicate that organic forms were more effective in their experiment, which, in their opinion, may result from the easier transport of chelated ions across cell membranes. Furthermore, anions can constitute additional carbon sources in cultivation and have a beneficial effect on the rhizosphere. Chloride ions derived from CaCl_2_ may, in turn, potentially induce salt stress and hinder calcium uptake. In addition to an increase in calcium content (Table 5), the authors observed a decrease in Mg content after the application of calcium chloride, an increase in Fe content after calcium citrate, a decrease in Na content with all forms used, and a rather small effect on Cu, Zn and potassium content. In the study by Zeng et al. [108], CaSO_4_ did not reduce the levels of any of the tested minerals in broccoli sprouts (Table 5). Some studies [104,108,109,110] assessed the effect of calcium or calcium and strontium while simultaneously subjecting germinating plants to salt stress. The results of analyses of the impact of additives used in the presence of salt stress on plants are important not only because of the possibility of modifying the composition of the products obtained, but also because of the frequently occurring soil conditions. The aforementioned studies observed a beneficial effect of calcium on increases in indicators such as polyphenol content and fresh product weight. In the presence of CaSO_4_, a mitigation of the decrease in glucosinolate content caused by salt stress was also observed [108].

## 4. Conclusions

Biofortification of deficient trace elements in microgreens and sprouts is a promising method of obtaining plant products with a very high content of selected minerals. The publications presented above indicate that microgreens and sprouts have a high capacity to accumulate components such as iodine, selenium, zinc, and calcium. However, there is a lack of analysis to determine the form in which the applied minerals occur in enriched plants and their bioavailability. This is significant because of the differences in the absorption of various forms of minerals by the human body. Due to the relatively small number of studies on the biofortification of sprouts and microgreens, there is also a lack of comprehensive information on the influence of genetic factors (species and varieties) on the utilisation of minerals added during cultivation. At the same time, the conditions used in the experiments differ in terms of whether the plants receive only the biofortifying component or also, for example, a nutrient solution, the time of growth, cultivation conditions such as lighting, the moment of fertiliser application and its form and concentration. The potential for reducing mineral losses during seed soaking before germination has not been sufficiently analysed. As a result, despite very encouraging results, it is difficult to systematise and establish detailed rules for the most effective biofortification with individual mineral components. Given the risk of excessive accumulation of micronutrients such as selenium, it is also important to develop precise biofortification protocols to ensure that levels of minerals remain safe for consumers. It also seems advisable to attempt to introduce other minerals such as magnesium or chromium. Research on this topic is currently lacking in the literature.

## Figures and Tables

**Table 2:** Results of iodine biofortification of sprouts and microgreens.

Raw Material	Treatment	Result	Reference
Pepper microgreens	Soaking seeds (2 h, 50 °C) in KI solutions: 0, 0.01, 1 mmol/L) with or without mixture of *Bacillus velezensis* (1.2 × 10^8^ cfu/mL)	↑ germination rate, fresh biomass, vitamin C and total phenolic content, post-harvest senescence delay for all treatments, ↑ iodine content with the exception of *B. velezensis* alone treatment	[49]
Tatsoi, coriander, green basil, purple basil microgreens	nutrient solution (0, 4, 8 μM of iodine as potassium iodide)	8 μM increases the iodine average concentration by 226.5%	[48]
Common buckwheat microgreens	Seed soaked (4 h) in 0 or 1000 mg/L of I (in the form of IO_3_^−^ iodate or I^−^ iodide) and other solutions	IO_3_^−^ ↑ I content (217 µg/g dw) compared to control (8.5 µg/g dw) and I^−^ (18 µg/g dw)	[61]
Swiss chard, rocket, pea and radish microgreens	KIO_3_ (0, 1.5 and 3 mg of I/L) in nutrient solution	Compared with the control, 1.5 and 3 mg/L of I resulted in 4.5 and 14-fold higher I levels in microgreens. The highest content in Swiss chard (3 mg/L)—865 μg I/100 g FW.	[62]
Tatsoi, coriander, green basil, purple basil microgreens	KI (0, 4, 8 µM) in nutrient solution	dose-dependent ↑ iodine content; for 8 µM 200.7, 118.2, 94, and 82.7 mg I/kg of dry weight, in tatsoi, coriander, purple basil, and green basil, respectively. For control: 101, 13.9, 51.8, 10.8 mg/kg, respectively	[13]
Yellow lupine, lentil, red and white clover, common vetch sprouts	Soaking (24 h) and watering (7 days) seeds in water or 6.5 mg/L potassium iodide solution	Soaking in solution and watering with water up to 10.4 μg I/100 g fw (yellow lupine), soaking in water and watering with solution up to 1026.7 μg I/100 g fw (white clover), soaking and watering in solution up to 857.1 μg I/100 g fw (white clover), controls up to 1.1 μg I/100 g fw (white clover)	[60]

Note: ↑ increase in content; Abbreviations: dry weight (dw); colony-forming unit (cfu).

**Table 4:** Results of iron biofortification of sprouts and microgreens.

Raw Material	Treatment	Result	Reference
Arugula, red cabbage, red mustard microgreens harvested 11 days after sowing	Iron sulfate heptahydrate (0, 10, 20, 40 mg Fe/L) in nutrient solution	↑ iron concentration in plants with increasing Fe concentration in nutrient solution up to 11.1–44.9 mg Fe/100 fw for 40 mg/L in nutrient solution. Iron addition influenced other mineral levels in a manner depending on the species	[79]
7-day common buckwheat sprouts	Elicitation by everyday immersion with 4 mM Na_2_SiO_3_ solution (SIL) or SIL and 0.5 mM Fe-EDTA solution	SIL-Fe caused ↓ Ca, K, Na, Cu, and Zn; a several-fold increase in Fe and Si content, and 20% lower fresh matter	[87]
12–day broccoli microgreens	Bacteria capped γ-Fe_2_O_3_ (68 nm) NPs, uncapped γ-Fe_2_O_3_ NPs, mineral γ-Fe_2_O_3_ NP precursor, uncapped γ-Fe_2_O_3_ with biofertilizer, mineral γ-Fe_2_O_3_ NP precursor with biofertilizer and others applied at days 1st, 6th, and 9th day of cultivation	Bacteria-capped γ-Fe_2_O_3_ NPs, uncapped γ-Fe_2_O_3_ NPs, mineral γ-Fe_2_O_3_ NP precursor, mineral ZnO NP precursor applied with biofertilisers, and uncapped γ-Fe_2_O_3_ NPs applied in conjunction with biofertiliser caused ↑ Fe content	[77]
14 days microgreens of purple kohlrabi, radish, pea Boogie, spinach	nutrient solution containing 1.5, 3.0 mg Fe /L (Librel FeDP7 chelate, 7% Fe)	Increasing the concentration of Fe caused ↑ Fe content in the kohlrabi, pea, spinach; ↓ Cu in all species, ↓ Zn in kohlrabi, pea, ↑ Mn in kohlrabi, spinach	[86]
5-day alfalfa, broccoli, radish sprouts	Seeds were soaked (7 h alfalfa, 5 h radish, 8 h broccoli) in Fe(III)-EDTA or Fe(III)-citrate solutions (10, 5.0, 2.5 mM)	↑iron concentration up to 1.8 times more than in controls	[72]
7-day soybean sprouts	seeds soaking (12 h) and watering with FeSO_4_ solutions (0, 5, 10, 15, 20, 25 mM FeSO_4_).	20–25 mM FeSO_4_—strong inhibition of germination, ↑ ferritin iron content, ↑ total iron content from 11 (0 mM FeSO_4_) to 276 (25 mM FeSO_4_) mg/100 g d.m.	[89]
Sunflower and water spinach microgreens	after seven days of germination an 8-day treatment period with FeSO_4_ solution (0, 0.1, 0.2, and 0.3 mM Fe), pH 4.5	↑ Fe content 2.69, 3.01, 2.76 times over sunflower control; ↑ Fe content 2.50, 2.64, and 3.87 times over the water spinach control; ↓ Ca and K in sunflower ↔ Ca and K in water spinach, no effect on Mg content	[88]

Note: ↑ increase in content; ↓ decrease in content; ↔ result not significantly different from control. Abbreviations: NPs—nanoparticles; dry weight (dw); Na_2_SiO_3_ solution (SIL).

**Table 5:** Results of calcium biofortification of sprouts and microgreens.

Raw Material	Treatment	Result	Reference
Broccoli microgreens	Soaking microgreens in 10mM CaCl_2_ water solution for 3 days	Accumulation of Ca in cotyledons in the form of Ca(II) hydrated complex	[103]
Broccoli microgreens	Spraying daily for 10 days with 0, 1, 10, 20 mM of calcium chloride water solutions	10 mM CaCl_2_ ↑ biomass, superoxide dismutase and peroxidase activities, tripled the calcium content, ↓ tissue electrolyte leakage	[102]
Peanut sprouts	15 mM CaCl_2_ treatment (alone or in combination with 150 µM MeJa or MeJa + 10mM LaCl_3_) during 5-day sprouting	CaCl_2_, CaCl_2_ + MeJa ↑ the sprouts’ length, ↑ resveratrol content and Ca^2+^ content	[105]
Common vetch sprouts	0.5, 1, 5 mM (Ca^2+^) solutions of calcium chloride, calcium formate, calcium acetate; 0.16, 0.33, 0.167 mM of calcium citrate as culture medium during 6-day hydroponic cultivation	↑ calcium content, the biggest for calcium acetate, followed by calcium formate, calcium citrate and calcium chloride; CaCl_2_ ↑ TPC	[106]
Djulis (Taiwanese quinoa) sprouts	1 week hydroponically grown sprouts were next 5 days treated with 5 mM CaCO_3_	↑ calcium content, chlorophyll content, SOD, CAT, APX, GR activity; ↓ H_2_O_2_ and MDA content ↓ K^+^ in roots; no significant effect on K^+^ in shoots	[111]
Barley sprouts	6 days germination with NaCl stress (60 mM)—control; among other combinations: NaCl + 0.4 mM CaCl_2_, or CaCl_2_ + melatonin +NaCl	↑ phenolic acid content, fresh weight, total calcium content, CAT, SOD and POD activity	[109]
Broccoli microgreens	2 days sprouts were until 9 day after sowing treated with distilled water (control), 80 mmol/L NaCl and/or 1.39 mmol/L CaSO_4_	NaCl ↓ yield, total glucosinolates, nitrate, ↑ Na, K, ↓ Ca, Mg, Zn; CaSO_4_ + NaCl ↑ yield, ↓ total glucosinolates, nitrate, ↓ P, Mg, ↑ S, Na, Ca; CaSO_4_ ↑ yield, total glucosinolates, ↔ nitrate, ↑ S, K, Ca	[108]
Soybean sprouts	4 days germination with NaCl stress (40 mM)—control; among other combinations: NaCl + 6 mM CaCl_2_, NaCl + CaCl_2_ + 5 mM LaCl_3_, NaCl + CaCl_2_ + 3-MP	↑ content of phenolic compounds, total isoflavonoids, calmodulin; NaCl + CaCl_2_, NaCl + CaCl_2_ + LaCl_3_ ↑ Ca^2+^ content; NaCl + CaCl_2_+ 3-MP ↓ Ca^2+^ content;	[104]

Note: ↑ increase in content; ↓ decrease in content; ↔ result not significantly different from control. Abbreviations: methyl jasmonate (MeJA), Lanthanum (III) trichloride (LaCl_3_)—a calcium channel blocker, Malondialdehyde (MDA), total polyphenol content (TPC); superoxide dismutase (SOD), catalase (CAT), ascorbate peroxidase (APX), and glutathione reductase (GR), 3-mercaptopropionate (3-MP)—inhibitor of GABA biosynthesis.

## Data Availability

The author has nothing to report.
